# Outbreak of paediatric myocarditis associated with parvovirus B19 infection in Italy, January to October 2024

**DOI:** 10.2807/1560-7917.ES.2024.29.48.2400746

**Published:** 2024-11-28

**Authors:** Marco Poeta, Cristina Moracas, Francesca Ippolita Calò Carducci, Claudio Cafagno, Danilo Buonsenso, Marco Maglione, Sofia Sgubbi, Cecilia Liberati, Elisabetta Venturini, Giuseppe Limongelli, Felice Nunziata, Laura Petrarca, Claudia Mandato, Claudia Colomba, Alfredo Guarino, Pediatric INF-ACT Network Study Group, Collaborators of the Paediatric INF-ACT Network Study Group, Andrea Lo Vecchio, Sandra Trapani, Anna Maria Musolino, Giovanni Meliota, Matteo Scalpelli, Antonietta Giannattasio, Amelia Licari, Daniele Donà, Emanuele Monda, Giulia Ranucci, Simona Marra, Antonio Guerriero, Valeria Garbo, Federica Pagano

**Affiliations:** 1 Department of Translational Medical Science, University of Naples “Federico II”, Naples, Italy; 2 Pediatric Infectious Disease Unit, Department of Maternal and Child health, University Hospital “Federico II”, Naples, Italy; 3 PhD National Program in One Health approaches to infectious diseases and life science research Department of Public Health, Experimental and Forensic Medicine, University of Pavia, Pavia, Italy; 4 Infectious Disease Unit, Bambino Gesù Children’s Hospital, IRCCS, Rome, Italy; 5 Infectious Diseases, Children's Hospital Giovanni XXIII, Azienda Ospedaliero Universitaria Consorziale Policlinico di Bari, Bari, Italy; 6 Department of Woman and Child Health and Public Health, Fondazione Policlinico Universitario A. Gemelli IRCCS, Rome, Italy; 7 Pediatric Emergency Department, Santobono-Pausilipon Children Hospital, Naples, Italy; 8 Department of Clinical, Surgical, Diagnostic and Pediatric Sciences, Fondazione IRCCS Policlinico San Matteo, Pavia, Italy; 9 Division of Pediatric Infectious Diseases, Department for Women's and Children's Health, University of Padua, Padua, Italy; 10 Infectious Diseases Unit, Meyer Children's Hospital IRCCS, Florence, Italy; 11 Inherited and Rare Cardiovascular Diseases, Department of Translational Medical Sciences, University of Campania “Luigi Vanvitelli”, Monaldi Hospital, Naples, Italy; 12 Department of Pediatrics, AORN Sant'Anna E San Sebastiano, Caserta, Italy; 13 Department of Maternal, Infantile and Urological Sciences, Sapienza University of Rome, Rome, Italy; 14 Department of Medicine, Surgery and Dentistry “Scuola Medica Salernitana”, University of Salerno, Salerno, Italy; 15 Department of Health Promotion, Mother and Child Care, Internal Medicine and Medical Specialties-University of Palermo, Palermo, Italy; 16 The members of the Pediatric INF-ACT Network Study Group are listed under Collaborators

**Keywords:** Myocarditis, children, Parvovirus B19, outbreak, infectious threat

## Abstract

Acute myocarditis has risen among paediatric patients in Italy, with 65 clinically suspected cases reported by 12 centres in 2024, 32 linked to parvovirus B19 (B19V) infection. In 11 cases, B19V was not ruled out despite a concurrent European outbreak. Twenty-nine children required intensive care; eight fatalities occurred. While effective for both severe B19V infection and myocarditis, intravenous immunoglobulins were given in only one-third of cases. These findings highlight the need for timely diagnosis, stronger surveillance, and standardised treatment protocols.

Acute myocarditis is a life-threatening condition in children, with viral infections being the most common cause [1]. Following the end of public health measures in response to the COVID-19 pandemic (including social distancing, mask-wearing, and school closures), an increase in the circulation of various bacterial and viral pathogens has been observed in several countries, often accompanied by higher numbers of severe infections [2]. Since the end of 2023, increases in detections of parvovirus B19 (B19V), a virus causing outbreaks every 3–4 years in high-income countries, have occurred in Europe [3]. Here, we describe 65 cases of paediatric myocarditis in Italy, of whom 32 were infected with B19V.

## Case definition

Acute myocarditis was defined according to the American Heart Association criteria [4]. Clinically suspected cases were individuals presenting signs and symptoms of heart failure, elevated troponin and/or electrocardiography/echocardiography abnormalities. All clinically suspected cases were included in the study. In some instances, cases were confirmed by cardiac magnetic resonance imaging (MRI).

The diagnosis of B19V infection was based on positive IgM serology, DNA amplification from blood samples, or positive DNA testing on endomyocardial biopsy.

## Inclusion of cases and data sources

The current investigation was conducted through a paediatric surveillance system established within the network of a European Union (EU)-funded National Recovery and Resilience Plan (NRRP) project, the *One Health Basic and Translational Actions Addressing Unmet Needs on Emerging Infectious Diseases* (INF-ACT) [5]. Information on cases was obtained retrospectively from a total of 12 paediatric centres that were involved in the INF-ACT surveillance of acute myocarditis. The centres were tertiary care facilities representative of the Italian paediatric hospitalised population and were distributed across the country, covering regions from the north to the south of Italy.

All clinically suspected acute myocarditis cases aged ≤ 18 years, who were hospitalised in the participating centres from January 2024 to October 2024 were included. These are further referred to as cases. Clinicians in the centres reported confirmatory MRI and virological examinations when available.

Demographic, time distribution, clinical symptoms, laboratory, imaging, and therapeutic data were collected from the 12 paediatric centres. To analyse epidemiological trends, the number of paediatric myocarditis cases in 2022 and 2023 was retrospectively collected from hospital discharge records of six of the 12 centres.

## Demographic characteristics and temporal distribution of cases

**Figure 1:**
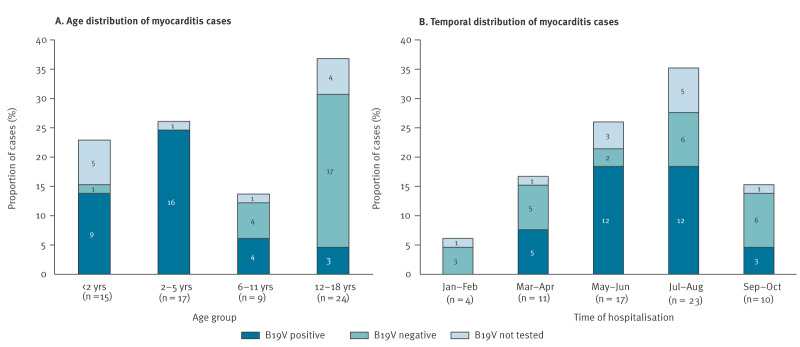
(A) Age and (B) temporal distribution of myocarditis cases according to B19V related testing, Italy, January−October 2024 (n = 65 cases)

From 1 January to 31 October 2024, a total of 65 children with clinically suspected myocarditis were hospitalised at 12 participating centres. Their median age was 6.1 years (interquartile range (IQR): 2.2–13.7). The most affected age group (n = 24, 37%) consisted of adolescents (13–18 years old) (Figure 1A), 39 (60%) were males and 26 (40%) were female. Almost two-thirds of cases (n = 40, 62%) were reported between May and August, with a peak in summer (July–August, n = 23) and an apparent decrease in cases starting in September (Figure 1B).

B19V: parvovirus B19.

In the six centres of the 12 centres, where data on past hospitalisations with myocarditis were available, 44 hospitalised cases were detected during the study period, suggesting an increase compared to 2022 when 24 cases were recorded and 2023 when there were 25 cases. 

## Clinical and laboratory features of cases

The medical history revealed a chronic disease/underlying condition in 10 cases: asthma (n = 2), thyroid dysfunction (n = 2), recurrent myocarditis (n = 1), inflammatory bowel disease (n = 1), cow's milk allergy (n = 1), neurological (n = 1), cardiological (n = 1) or urological (n = 1) malformations.

Fever, defined as body temperature ≥ 38 ˚C (40%), asthenia (38.5%) and chest pain (36.9%) were the most prevalent symptoms, followed by dyspnoea (30.8%) and vomiting (27.7%). Cardiogenic shock was observed in 10 cases (15.4%). All 65 children showed abnormal elevation of myocardial enzymes (high sensitive (hs) Troponin T or I > 20 pg/mL), and those with abnormal ventricular function (n = 29) exhibited increased levels of brain natriuretic peptide (BNP) (> 100 pg/mL). Additionally, 35 children (53.8%) had elevated inflammatory markers (C-reactive protein > 5 mg/L) (Table).

**Table:** Demographic, clinical, and laboratory features and main treatments of cases of myocarditis, Italy, 1 January–31 October 2024 (n = 65 cases)

Characteristics of all cases	Study population (n = 65)	
	Number^a^	%^b^
Demographic features		
Age at admission in years, median (IQR)	6.1 (2.2–13.7)	
Male sex	39	60.0
Chronic diseases	10	15.4
Clinical features		
Fever (body temperature ≥ 38 ˚CI)	26	40.0
Asthenia	25	38.5
Chest pain	24	36.9
Dyspnoea	20	30.8
Vomiting	18	27.7
Cardiogenic shock	10	15.4
Diarrhoea	9	13.8
Palpitation	8	12.3
Abdominal pain	8	12.3
Rash	7	10.8
Syncope	4	6.2
Cough	2	3.1
Length of hospital stay, mean days (SD)	15.0 (12.7)	
Biochemical parameters		
Haemoglobin in g/dL, mean (SD) (norm: 11–16)	11.9 (2.1)	
WBC in cells/µL, mean (SD) (norm: 4.5–11.0)	11,325 (4,937)	
Neutrophils in cells/µL, mean (SD) (norm: 1.8–7.0)	9,482 (13,947)	
Lymphocytes in cells/µL, mean (SD) (norm: 1.0–4.8)	3,448 (3,127)	
Platelet count, x10^3^ cells/µL, mean (SD) (norm: 150–450)	311 (139)	
CRP in mg/L, mean (SD) (norm: 0–5)	26.3 (51.3)	
Procalcitonin in ng/mL, mean (SD) (norm: 0–0.5)	3.7 (12.5)	
Positive CRP	35	53.8
Positive hs-Troponin I or T	65	100
hs-Troponin I in pg/mL, mean (SD) (norm: 0–16)	1,677.6 (2,883.2)	
hs-Troponin T in pg/mL, mean (SD) (norm: 0–14)	944.1 (1,338.9)	
BNP in pg/mL, mean (SD) (norm: 0–100)	18,079.7 (23,972.3)	
CK-MB, ng/mL, mean (SD) (norm: 0–3.4)	24.7 (33.4)	
AST in IU/L, mean (SD) (norm: 10–50)	86.1 (177.4)	
LDH in IU/L, mean (SD) (norm: 190–320)	411.0 (330.5)	
ECG features, ratio^c^ and %		
Abnormal findings	37/60	36.8
Repolarisation abnormalities	15/60	25.0
ST-T changes	13/60	21.6
Low QRS voltage	8/60	13.3
Prolonged QTc	3/60^a^	5
Echocardiographic features		
Abnormal findings	44	67.7
LVEF < 50%	29	44.6
Wall motion anomalies	27	41.5
LV enlargement	20	30.8
Valvular regurgitation	19	29.2
Thickened myocardium	10	15.4
Pericardial effusion	7	10.8
Case status, number		
Clinically suspected	65	
− Of whom confirmed (MRI with or without biopsy)	18	

AST: Aspartate aminotransferase; BNP: brain natriuretic peptide; CK-MB: creatine phosphokinase-MB; CRP: C-reactive protein; ECG: electrocardiogram; hs-Troponin: High-sensitivity troponin; IQR: Interquartile range; IU: international units; LDH: Lactate Dehydrogenase; LV: Left ventricular; LVEF: Left ventricular ejection fraction; norm: normal values; SD: standard deviation; WBC: white blood cells.

^a^ Cells in the column show the number of cases, unless the row heading specifies another type of value.

^b ^Cells in the column show the percentages of cases, unless the row heading specifies another type of value.

^c ^The denominator was 60 instead of 65 because for five cases the ECG features were not available.

Thirty-seven children showed abnormal electrocardiograms (ECGs), with nonspecific repolarisation abnormalities and ST-T changes being the most common findings. Of 60 children with available information on ECG features, 29 (44.6%) had a left ventricular ejection fraction (LVEF) < 50%, as determined by echocardiography. Wall motion anomalies were observed in 27 cases (41.5%) and left ventricular enlargement in 20 cases (30.8%) (Table). Cardiac magnetic resonance imaging (MRI) was performed in 18 of 65 children, confirming the clinically suspected diagnoses and showing varying degrees of myocardial inflammation. Endomyocardial biopsy was obtained in three cases for histological confirmation.

Twenty-nine children (45%) required intensive care unit (ICU) admission, with a median age of 2.2 years (IQR: 1–4.3). One was a candidate for a heart transplant. Eight children (12%) died, with a median age of 1.4 years (IQR: 0.9–9). Death occurred from 12 hours to 19 days of intensive care. 

## Serology and PCR results for infectious pathogens

**Figure 2:**
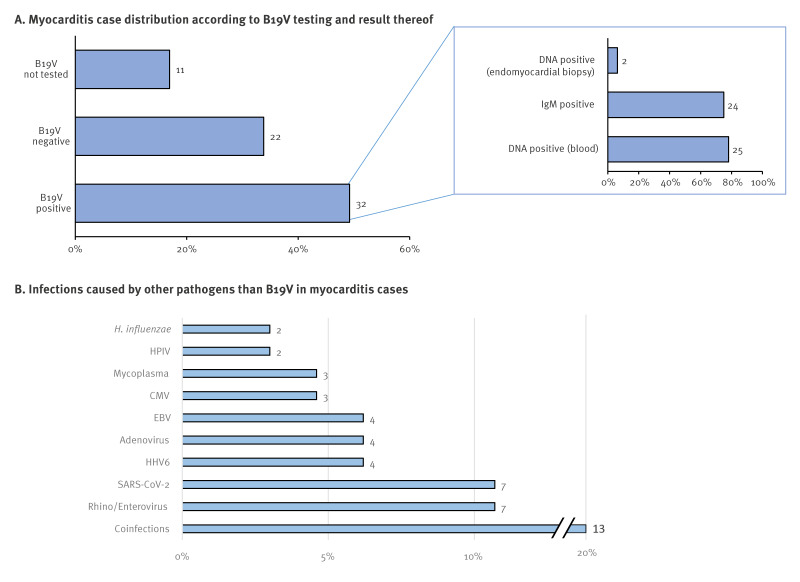
(A) Distribution of cases according to parvovirus B19 testing and results thereof as well as (B) other infectious aetiologies found for myocarditis cases, Italy, 1 January–31 October 2024 (n = 65 cases)

B19V: parvovirus B19; CMV: cytomegalovirus; EBV: Epstein–Barr virus; *H. influenzae*: *Haemophilus influenzae*; HHV6: human herpesvirus 6; HPIV: human parainfluenza virus; SARS-CoV-2: severe acute respiratory syndrome coronavirus 2.

An aetiological cause was identified in 47 cases, with the most frequently identified pathogen being B19V, diagnosed in 32 of the total 65 cases (49.2%) (Figure 2A). B19V was not tested in 11 (16.9%) cases. Other identified infectious agents are listed in Figure 2B. A coinfection (with or without B19V) was recorded in 13 (20%) cases. Two of three biopsy cases showed positive virological findings for B19V in the myocardium, with one case co-infected with human herpesvirus 6 (HHV6), and one case co-infected with HHV6 and Epstein–Barr virus (EBV). In the biopsy of the third case, virological examinations did not find any virus. Of 32 cases requiring ICU admission 20 had B19V infection, seven were negative and five not tested. Among the eight cases who died, four tested positive for B19V and four were not tested.

In panel A, summing patients in each diagnostic method category yields a higher number than the B19V positive because many children tested positive for both DNA and serology.

## Treatment of cases

**Figure 3:**
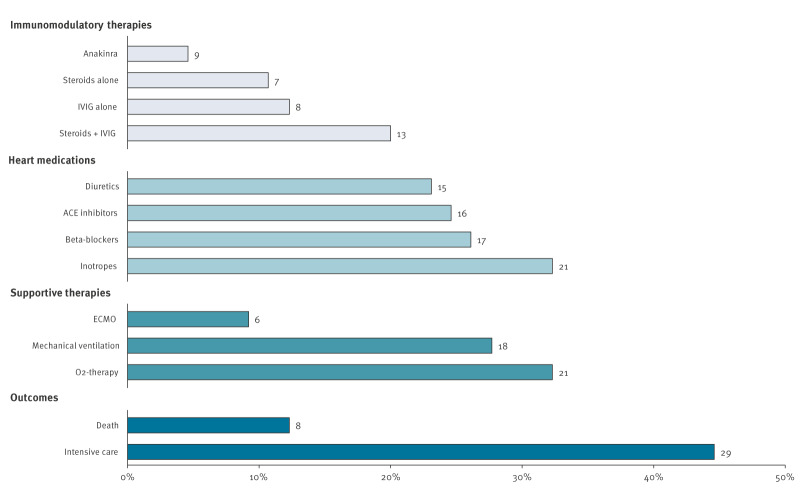
Therapies and outcomes of hospitalised cases of myocarditis, Italy, 1 January–31 October 2024 (n = 65 cases)

ACE: angiotensin-converting-enzyme; ECMO: extracorporeal membrane oxygenation; IVIG: Intravenous immunoglobulin.

Twenty-eight (43%) children received immunomodulatory therapy, including steroids alone (n = 7), intravenous immunoglobin (IVIG) alone (n = 8), or a combination of both (n = 13). Nine cases received the interleukin-1 receptor antagonist Anakinra. Non-steroidal anti-inflammatory drugs (NSAIDs) or colchicine were administered in presence of myopericarditis (n = 23, 35%). Cardiologic medications and supportive therapies are listed in Figure 3.

## Discussion

Following the COVID-19 pandemic, a resurgence of various infectious diseases occurred in many countries [2]. This led to the hypothesis that non-pharmaceutical measures taken to mitigate severe acute respiratory syndrome coronavirus 2 (SARS-CoV-2) transmission during the pandemic may have temporarily reduced people’s exposure to many communicable pathogens, resulting in an 'immunity gap' [2]. Once measures were lifted, this gap may have contributed to larger outbreaks and a greater number of severe infections, even when caused by common pathogens and affecting immunocompetent individuals [2]. 

Since the beginning of 2024, considerably more hospitalised cases of clinically suspected myocarditis have been noted in Italy, particularly during the summer months. The rise in cases coincides with an increase of B19V activity in the United States [6] as well as in Europe [3], where a B19V outbreak appears to be ongoing. While usually, B19V results in frequent asymptomatic or mild infections, especially in children, during the current B19V European outbreak, an increase of severe infections has been observed in several countries, including the cases of myocarditis in Italy [7].

There is mounting evidence pointing to B19V as a current primary aetiological agent of acute myocarditis [1]. According to a systematic review by Krych et al., this virus has been detected in one fourth of paediatric cases [8] and has a cardiotropic nature. Primarily targeting endothelial cells rather than cardiomyocytes, B19V promotes a prolonged inflammatory response responsible for chronic myocardial damage [9], potentially leading to dilated cardiomyopathy and progressive heart failure [8].

In our study population, half of the children with acute myocarditis presented with B19V infection, identified through non-invasive methods (serology and DNA blood testing). In three cases, an endomyocardial biopsy was performed for histological confirmation and virological analysis. This identified a B19V in two cases, one of whom was co-infected HHV6 and the other with HHV6 and EBV. Additionally, 17% of children were not tested against B19V, leaving the true incidence of B19V potentially underestimated in this population. Furthermore, considering only the children tested for B19V, the incidence of infection is higher (60%).

In our cohort, a high number of patients were immunocompetent adolescent males (n = 24), consistent with previous findings [1]. Why B19V causes myocarditis in some cases but not in others remains unclear. Recent studies suggest that a genetic predisposition may play a role [10], and interestingly, one child in our series experienced recurrent myocarditis. Therefore, close monitoring post-recovery and screening for genetic cardiomyopathies (next generation sequencing panels) should be considered to identify individual susceptibility to recurrent myocarditis or cardiomyopathy.

Immunosuppressive drugs and IVIG are associated with better outcomes in paediatric viral myocarditis [11]. In our study, only 32% of patients received IVIG, either alone or in combination with steroids. Combining IVIG with high-dose steroids improves left ventricular function without significant adverse events [12], and fewer deaths have been reported in children treated with IVIG compared with those treated with steroids alone [13]. Additionally, IVIG is an effective treatment for severe B19V infection, although its use varies across centres. Given its dual role in the treatment of both severe B19V infection and viral myocarditis, early initiation of therapy should be considered, even in the absence of a confirmed aetiology, especially during outbreaks. In addition, biological drugs targeting the interleukin-1 pathway have shown promising results in severe B19V-related myocarditis [9] and were used in nine children in our series, warranting further study.

Healthcare providers should remain vigilant for severe B19V manifestations, including myocarditis, even in otherwise healthy children and adolescents. B19V myocarditis has high mortality and morbidity, with a 37.5% mortality rate and only 26% of patients achieving full cardiac recovery [14]. In our series, eight children died, 29 required intensive care, and one was listed for a heart transplant, highlighting the need for rapid identification and targeted treatment. Screening for genetic cardiomyopathies [10] should also be considered for relatives in presence of severe cases or deaths.

The current study has some limitations. One is that only half of the centres had retrospective data available to check epidemiological trends compared to previous years. Another is that diagnostic and treatment protocols varied across centres, which may have affected the assessment of the incidence of B19V and potentially led to an underestimation of myocarditis cases affected by this virus. This highlights the need for higher awareness among physicians of the importance of testing myocarditis cases for infectious pathogens.

## Conclusions

Historically, public health efforts to monitor B19V have been limited due to the perceived benign nature of this infection, with routine surveillance absent and reporting non-mandatory. However, the recent increase in cases and the rise of potentially associated myocarditis underline the need for proactive vigilance at least during epidemic periods. The testing of B19V in patients with myocardial injury should be incorporated into paediatric management protocols and it should be recommended to clinicians to promptly start effective therapies adapted for viral/B19V infections. Conversely, screening of myocarditis could be performed in hospitalised children with B19V infection and active treatment considered.
